# Effect of L-arginine supplement on liver regeneration after partial hepatectomy in rats

**DOI:** 10.1186/1477-7819-10-99

**Published:** 2012-05-31

**Authors:** Jun An, Koji Tsunekawa, Yoshiharu Shimomura, Shunsuke Kazama, Naohisa Ishikawa, Toshiaki Nonami, Satoru Sugiyama

**Affiliations:** 1 Division of Gastroenterological Surgery, Department of Surgery, Aichi Medical University School of Medicine, Nagakute, Aichi Pref 480-1195, Japan; 2 Department of Surgery, Dalian Municipal Friendship Hospital, No. 8, San Ba Plaza Zhongshan District, Dalian, Liaoning 116001, China; 3 Department of Pharmacology, Aichi Medical University School of Medicine, Nagakute, Aichi Pref 480-1195, Japan; 4 Institute of Applied Biochemistry, Oshima-cho, Chikusa-ku, Nagoya, Aichi Pref 464-0833, Japan; 5 Department of Applied Molecular Biosciences, Graduate School of Bioagricultural Sciences, Nagoya University, Furo-cho, Chikusa-ku, Nagoya, Aichi Pref 464-8601, Japan; 6 Masuko Memorial Hospital, 35-28 Takehashicho, Nagoya, Nakamura-ku 453-0016, Japan

**Keywords:** Amino acid, L-arginine, L-glutamine, Hepatectomy, Liver regeneration

## Abstract

****Background**:**

Nitric oxide (NO) has been reported to be a key mediator in hepatocyte proliferation during liver regeneration. NO is the oxidative metabolite of L-arginine, and is produced by a family of enzymes, collective termed nitric oxide synthase (NOS). Thus, administration of L-arginine might enhance liver regeneration after a hepatectomy. Another amino acid, L-glutamine, which plays an important role in catabolic states and is a crucial factor in various cellular and organ functions, is widely known to enhance liver regeneration experimentally. Thus, the present study was undertaken to evaluate the effects of an L-arginine supplement on liver regeneration, and to compared this with supplementation with L-glutamine and L-alanine (the latter as a negative control), using a rat partial hepatectomy model.

****Methods**:**

Before and after a 70% hepatectomy, rats received one of three amino acid solutions (L-arginine, L-glutamine, or L-alanine). The effects on liver regeneration of the administered solutions were examined by assessment of restituted liver mass, staining for proliferating cell nuclear antigen (PCNA), and total RNA and DNA content 24 and 72 hours after the operation.

****Results**:**

At 72 hours after the hepatectomy, the restituted liver mass, the PCNA labeling index and the DNA quantity were all significantly higher in the L-arginine and L-glutamine groups than in the control. There were no significant differences in those parameters between the L-arginine and L-glutamine groups, nor were any significant differences found between the L-alanine group and the control.

****Conclusion**:**

Oral supplements of L-arginine and L-glutamine enhanced liver regeneration after hepatectomy in rats, suggesting that an oral arginine supplement can clinically improve recovery after a major liver resection.

## **Background**

The result of extensive hepatectomy has been greatly advanced through operative techniques and perioperative management. That strategy has been maintained by the capacity for liver regeneration. Under normal conditions, the basal hepatocyte proliferation rate is low, and mitosis seldom occurs. However, after an event involving a substantial loss of hepatic tissue such as that found in severe viral hepatitis or a major hepatectomy, cell division leads to intense tissue growth until regeneration occurs, with the energetic and synthesis capacity being essentially maintained. This regenerative process is accompanied by compensatory cellular hyperplasia and hypertrophy, and is controlled and mediated by nutrients and growth factors. If liver regeneration after major hepatectomy could be enhanced artificially, the cases in which hepatectomy for liver cancers can be used would be extended, with improved patient prognoses.

Nitric oxide (NO) is known to be a multifunctional mediator, regulating blood pressure, gene expression, apoptosis, and mitogenesis in essentially every organ and tissue [1-3]. NO is enzymatically synthesized from L-arginine (L-Arg) by three nitric oxide synthase (NOS) isoforms: neuronal (n)NOS, inducible (i)NOS and endothelial (e)NOS. eNOS is a crucial mediator of vascular tone and blood flow, and plays major roles in liver physiology and pathophysiology [4]. NO has been reported to reduce organ injury and enhance liver regeneration by experimentally modulating hepatic macrohaemodynamics [5].

Recently, there have been studies assessing whether delivery of a specific amino acid can improve patient outcome, as some amino acids are precursors of many important biologic compounds essential for a normal functioning of the human organism [6]. Furthermore, these supplements can be taken orally to provide effective clinical availability. There have been no reports describing whether or not L-Arg enhances liver regeneration after hepatectomy. In the present study, we evaluated the effect of oral administration of L-Arg on liver regeneration after hepatectomy in rats.

## **Methods**

### **Rat hepatectomy model**

The study was approved by the Animal Care Committee of Aichi Medical University.

Seven-week-old male Wistar rats weighing between 170 and 190 g were purchased (SLC, Shizuoka, Japan) and habituated to the laboratory animal room for 1 week. All rats received humane care. They were kept in a climate-controlled room under 12-hour light/dark cycles with free access to food and tap water throughout the studies.

The rats were randomized into four groups (n = 12 in each group): control, L-Arg, L-glutamine (L-Gln) and the negative control L-alanine (L-Ala) (all amino acids from Wako, Osaka, Japan). Each group was divided into two subgroups (n = 6 in each subgroup) according to sample collection time (24 and 72 hours after hepatectomy). 10% L-Arg, 10% L-Gln or 10% L-Ala (1 ml/100 g body weight) was administered orally to the rats 15 minutes before a partial hepatectomy was carried out. The control group received no solution.

Under pentobarbital anesthesia (50 mg/kg body weight, administered intraperitoneally.), the abdomen of each rat was opened, and the median and left lobes of the liver were removed, using the standard Higgins and Anderson technique [7]. Rats in the control group underwent abdominal incision only.

Each rat in the treatment groups was also given the same amino acid after the surgery; the amino acid was added to the drinking tap water at a concentration of 1%.

At 24 and 72 hours after hepatectomy, the tissue used for the following experiments was collected. After each rat was anesthetized with pentobarbital sodium (50 mg/kg body weight intraperitoneally), the rats were killed.

### **Sample collection**

Small liver samples were collected from each rat, then frozen immediately and stored in liquid nitrogen until use for total DNA and RNA assays.

After the rats were killed, the blood was removed with saline, then a 4% paraformaldehyde solution (Wako, Osaka, Japan) dissolved in phosphate buffer was infused into the circulation via the left ventricle. The liver tissues were removed and fixed in 4% paraformaldehyde solution for 1 day. They were then hydrated through a graded alcohol and xylene series and embedded in paraffin wax. The samples were cut into sections 3 μm thick.

### **Restituted liver mass**

At the time of partial hepatectomy, the entire resected portion of the liver (designated B) was weighed. This weight was taken to be 30% of the total pre-hepatectomy liver weight, thus enabling calculations of the total liver weight (A). Once the rats were killed, the remaining liver portion (C) was excised and weighed. These data were expressed as a percentage of the ratio of remnant liver weight divided by the calculated total pre-hepatectomy weight and multiplied by 100 to yield a percentage restitution of liver mass [8]: Restituted liver mass (%) = [C-(A-B)/A] × 100.

### **Staining for proliferating cell nuclear antigen**

Hepatocyte regeneration was analyzed using immunohistochemical detection of proliferating cell nuclear antigen (PCNA) with a commercial kit (LSAB2 Kit; Dako, Kyoto, Japan). The sections were blocked against endogenous peroxidase using 3% H_2_O_2_ for 10 min, and rinsed in 0.01 mol/l TBS (50 mmol/l Tris–HCl, 0.15 mol/l NaCl, pH 7.6). Sections were incubated at 4°C overnight with primary mouse monoclonal anti-PCNA antibody (Dako, Kyoto, Japan) diluted 100 times in 0.01 mol/l PBS. Nuclear staining was performed using hematoxylin. Five fields per slide at 100× magnification were randomly selected, and the total hepatocytes per field were counted. The mean percentages of PCNA-positive hepatocytes were calculated and compared between the different groups.

### **Total RNA and DNA assays**

The liver tissue that had been stored in liquid nitrogen was removed and ground to a semi-powder at the same (liquid nitrogen) temperature. Total RNA and genomic DNA were then extracted from the semi-powdered tissue using commercial products (SV Total RNA Isolation System and Wizard SV Genomic DNA Purification System, respectively; both Promega Co., Madison, WI, USA), following the manufacturer’s instructions. The total RNA and genomic DNA content were determined by spectrophotometry at 260 nm.

### **Statistical analysis**

Data (given as mean ± standard error of the mean) were examined for significance using Kruskal-Wallis one-way analysis of variance, followed by Scheffé's method, and significance was set at *P* < 0.05.

## **Results**

### **Restituted liver mass**

**Figure 1:**
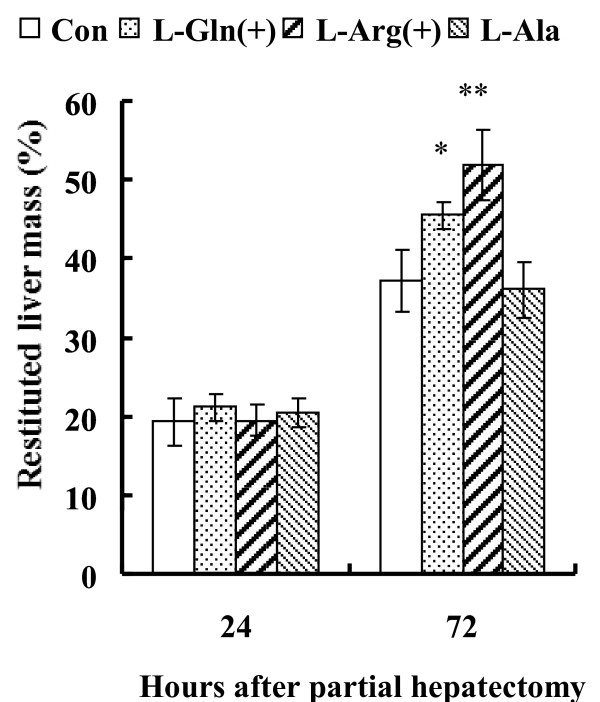
**Restituted liver mass at 24 and 72 hours after partial hepatectomy.** No significant differences in liver mass were evident at 24 hours after rats in each group underwent partial hepatectomy. There were significantly higher values found for the restituted liver mass in the L-Arg and L-Gln groups than in the control and L-Ala groups at 72 hours after partial hepatectomy. There were no significant differences in the restituted liver mass between the L-Arg and L-Gln groups. *: *P* < 0.05 and **: *P* < 0.01, compared with controls (n = 6 each).

No significant differences in liver mass were evident in any group at 24 hours after partial hepatectomy. There were significantly higher levels of restituted liver mass in the L-Arg (52.3 ± 4.2%; *P* < 0.01) and L-Gln (45.5 ± 1.7%; *P* < 0.05) groups than in the control (37.3 ± 4.1%) and L-Ala (36.0 ± 3.0%) groups at 72 hours after partial hepatectomy (Figure 1). There were no significant differences in restituted liver mass between the L-Arg and L-Gln groups.

### **Immunohistochemical staining results for proliferating cell nuclear antigen**

**Figure 2:**
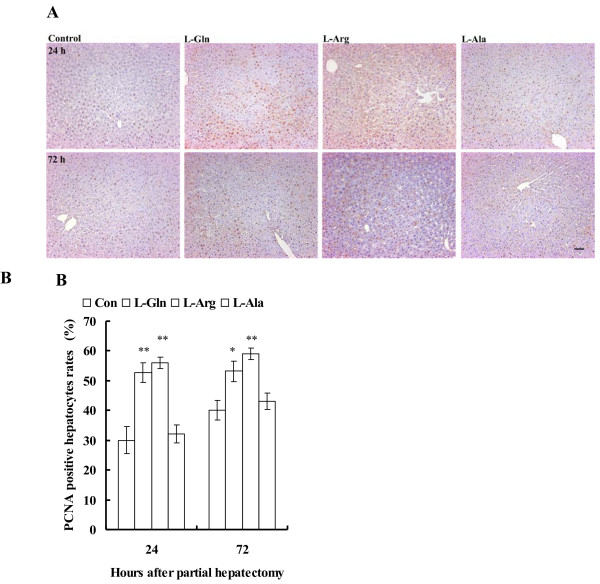
**Proliferating cell nuclear antigen (PCNA) immunohistochemical staining in liver at 24 and 72 hours after partial hepatectomy.** Representative photographs of PCNA immunohistochemical staining in each group were shown (original magnification × 100). Hepatic regenerative activity, expressed as the PCNA labeling index (%), was significantly improved in the L-Arg and L-Gln groups versus the control, but remained unchanged in the L-Ala group at 24 hours after partial hepatectomy. The L-Arg and L-Gln groups were also significantly improved compared with the control and L-Ala groups at 72 hours after partial hepatectomy. There were no significant differences in the PCNA labeling index between the L-Arg and L-Gln groups. * *P* < 0.05 and ** *P* < 0.01, compared with controls (n = 6 each).

Representative photographs of PCNA immunohistochemical staining in each group are shown in Figure 2A. Hepatic regenerative activity, expressed as the PCNA labeling index (%), was significantly improved in the L-Arg (56.1 ± 1.9%; P < 0.01) and L-Gln (52.8 ± 3.2%; *P* < 0.01) groups versus the control (30.0 ± 4.5%), but remained unchanged in the L-Ala (32.0 ± 3.0%) group at 24 hours after partial hepatectomy. Moreover, the L-Arg (59.0 ± 1.9%; *P* < 0.01) and L-Gln (53.1 ± 3.5%; P < 0.05) groups were also significantly improved compared with the control (40.1 ± 3.4%) and the L-Ala (43.1 ± 2.7%) groups at 72 hours after partial hepatectomy (Figure 2B). There were no significant differences in the PCNA labeling index between the L-Arg and L-Gln groups.

### **Total RNA and DNA assay**

Both the total RNA and the genomic DNA were measured as milligrams per gram of liver tissue. For the genomic DNA levels, no significant differences were detected at 24 hours after partial hepatectomy in each group, but at 72 hours, significantly increased levels of genomic DNA were found in the L-Arg (*P* < 0.05) and L-Gln ( *P* < 0.05) groups compared with the control group (Figure 2A). There were no significant differences in the DNA levels between the L-Arg and L-Gln groups. The DNA level in the L-Ala group also increased, but this was not significant.

**Figure 3:**
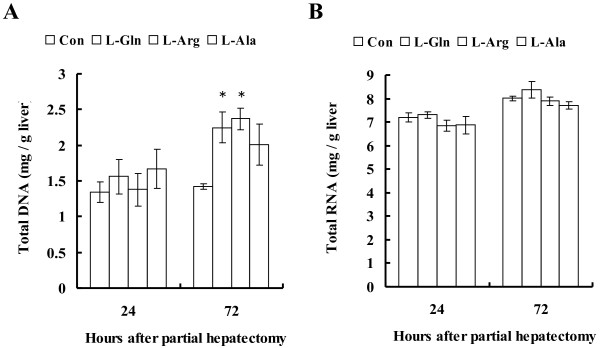
**Quantification of genomic DNA and total RNA at 24 and 72 hours after partial hepatectomy.** (**A**) No significant differences in the genomic DNA levels were found at 24 hours after partial hepatectomy in each group. However, at 72 hours after partial hepatectomy, significantly increased levels of genomic DNA were found in the L-Arg and L-Gln groups compared with the control group. There were no significant differences in DNA levels between the L-Arg and L-Gln groups. The DNA level in the L-Ala group also tended to increase, but not significantly. (**B**) No significant differences were found in total RNA levels between the control and the experimental groups at either 24 or 72 hours after a partial hepatectomy. * *P* < 0.05 compared with each control (n = 6 each).

For total RNA levels, no significant differences were found between the control and the experimental groups at either 24 or 72 hours after the partial hepatectomy (Figure 3).

## **Discussion**

Amino acids are the essential building blocks of all mammalian cells. In addition to their role in protein synthesis, amino acids also play an important role as energy fuel, precursors of a variety of metabolites, and as signaling molecules. Recently, there has been interest in assessing whether delivery of specific amino acids can improve patient outcome under various pathological conditions [6]. Gln is the most abundant amino acid in human muscle and plasma. Apart from providing nitrogen for protein synthesis, Gln is a precursor for nucleic acids, nucleotides, and the major antioxidant glutathione [9]. Recent data also indicate that Gln functions as a signaling molecule [10], particularly under catabolic conditions. Yoshida *et al.*[11] reported beneficial effects of Gln on DNA and on the protein synthesis of remnant liver in the 70% rat hepatectomy model. Since then, similar findings have been reported by a number of investigators [12,13]. In the present study, we evaluated the effects of L-Gln on liver regeneration, as measured by a restituted liver mass, the PCNA labeling index, and total DNA, and found basically equivalent results. Although Gln was administered intravenously in previous reports, we found oral administration of L-Gln to be similarly beneficial. Because oral supplementation of L-Gln is considered safer than intravenous infusion, this may be a better choice for clinical studies.

NO plays an important role in vasodilatation and in increasing blood flow by signaling smooth muscles to relax. A beneficial effect of NO on liver regeneration through an increase in hepatic arterial flow has been reported [5]. Studies have also shown that NO derived from eNOS regulates sinusoidal endothelial cell proliferation [14] and hepatocyte proliferation [4]. Carnovale *et al.*[15] showed that the hepatocyte iNOS level was increased after hepatectomy, and that NO derived from iNOS enhanced hepatic DNA synthesis, which had formerly been blocked by inhibiting iNOS expression. Observations of impaired liver regeneration and hepatocyte survival in iNOS knockout mice provide additional proof for the importance of NO-mediated signaling in liver regeneration [16]. It has also been shown that nitric oxide production contributes to hepatocyte proliferation in normal juvenile rats [17]. Although the mechanism of NO contribution to hepatocyte proliferation is not yet fully understood, it is known that NO derived from both eNOS and iNOS enhances liver regeneration.

Arg supplementation has been identified as advantageous in wound healing in experimental animals and humans [18,19]. The beneficial effects of Arg supplementation on wound healing have been attributed to enhanced synthesis of NO by NOS. NO is synthesized by both parenchymal and nonparenchymal cells of the liver [20]. The aim of the present study was to clarify whether direct administration of L-Arg has any effect on liver regeneration. We found that increases in restituted liver mass enhanced by L-Arg and L-Gln supplementation were associated with increases in the quantity of total hepatic DNA. Both restituted liver mass and DNA quantity were significantly higher in the L-Arg and L-Gln groups than in the control, suggesting that the hepatic regeneration was accompanied by cell proliferation. However, the hepatic regeneration was not accompanied by increases in the total RNA quantity, suggesting that the rate of protein synthesis might be in steady state. L-Ala was used as the negative control to match the energy and amino acid supply to L-Arg or L-Gln [21]. The significant differences found between L-Arg/L-Gln and L-Ala in the present study also reveals the specific functions of L-Arg and L-Gln in improving liver regeneration beyond the functions of simple nutrients. One report has shown different effects of L-Arg and L-Gln on liver regeneration in rats receiving total parenteral nutrition after hepatectomy [22]. To reveal the different mechanisms between L-Arg and L-Gln, another study containing an experimental group given both L-Arg and L-Gln together might be useful. Further study is warranted.

In our study, we found that L-Arg seems to have a similar potential for liver regeneration to that of L-Gln. Both amino acids proved effective even when orally administered, which is likely to facilitate clinical use. In our study, we gave rats oral 10% L-Arg (1 ml/100 g body weight) before hepatectomy and also administered it after the operation by adding it to tap water at a concentration of 1%. Witte *et al. *[23] reported that although the doses of Arg (20–50 mg/100 g/day) must be considered pharmacological, there is a notable lack of toxic side-effects. In light of their knowledge, our doses were two to five times higher on the operation day and about twice as high afterwards. However, when excessive amino acids are administered to patients with an impaired liver function such as liver cirrhosis, the increased ammonia levels can result in hepatic encephalopathy. Therefore, both the potential risks and benefits of L-Gln or L-Arg must be considered before treatment.

## **Conclusion**

In the present study, we found that both L-Arg and L-Gln enhanced liver regeneration after 70% rat hepatectomy. These results may contribute to the treatment of patients in need of major liver resection.

## Abbreviations

Ala, Alanine; Arg, Arginine; eNOS, Endothelial nitric oxide synthase; Gln, Glutamine; iNOS, Inducible nitric oxide synthase; nNOS, Neuronal nitric oxide synthase; NO, Nitric oxide; NOS, Nitric oxide synthase; PBS, Phosphate-buffered saline; PCNA, Proliferating cell nuclear antigen.

## Competing interests

The authors declare that they have no competing interests.

## Authors’ contributions

TK carried out design of the study and drafted manuscript. JA carried out experimental procedures and data analysis. KT, YS, SK carried out data analysis and reviewed the paper. NI, TN and SS guaranteed the whole study. All authors read and approved the final manuscript.
